# CDK4/6 Inhibitors in Hormone Receptor-Positive Metastatic Breast Cancer: Current Practice and Knowledge

**DOI:** 10.3390/cancers12092480

**Published:** 2020-09-01

**Authors:** Debora de Melo Gagliato, Antonio C Buzaid, Jose Manuel Perez-Garcia, Antonio Llombart, Javier Cortes

**Affiliations:** 1 Centro Oncológico Antonio Ermírio de Moraes, Beneficência Portuguesa de São Paulo, 01323001 São Paulo, Brazil; dgagliato@gmail.com (D.d.M.G.); buzaidac@yahoo.com.br (A.C.B.); 2 Centro Oncológico Hospital Israelita Albert Eisntein, 05652900 São Paulo, Brazil; 3 Baselga Institute of Oncology, Quiron University Hospital Barcelona, 08023 Barcelona, Spain; jose.perez@medsir.org (J.M.P.-G.); antonio.llombart@medsir.org (A.L.); 4 Ramon y Cajal University Hospital, 28034 Madrid, Spain; 5 Vall d’Hebron Institute of Oncology, 08035 Barcelona, Spain

**Keywords:** metastatic breast cancer, CDK4/6 inhibitors, hormone receptor-positive breast cancer

## Abstract

Treatment paradigms in advanced hormone receptor (HR)-positive breast cancer were substantially transformed with cyclin-dependent kinase 4 and 6 inhibitors (CDK4/6i) approval. The addition of these drugs to endocrine treatment profoundly improved progression-free and overall survival. Additionally, other important endpoints, such as the response rate, time to chemotherapy, and a delay in quality of life deterioration, were positively impacted by CDK4/6 inhibitors’ addition to the treatment of advanced HR-positive breast cancer. This review article will summarize current knowledge on CDK4/6 inhibitors in clinical practice for advanced HR-positive metastatic breast cancer, as well as describe recent efforts to more precisely characterize mechanisms of sensitivity and resistance to these drugs, both on the molecular and clinical characterization level.

## 1. Introduction

In breast cancer (BC), a variety of studies previously identified alterations in cell cycle regulators that can majorly impact tumor progression and development [1].

Activation of cyclin-dependent kinases (CDKs) enables cell cycle progression, representing the hallmark of cancer pathological development. The most important drivers of cell cycle proliferation are settled by CDK4/6 [1]. Crosstalk between cyclin D, CDK4/6, retinoblastoma-associated protein 1 (RB1), and estrogen receptor (ER) signaling occurs as a dynamic process, which can ultimately culminate in cell proliferation.

ER signaling leads to upregulation of cyclin D mRNA, as well as protein expression. In turn, cyclin D can activate both CDK4 and CDK6, which through phosphorylation of RB1, and release of transcription factor E2F, and promote cell cycle progression from the mitosis phase G1 to S, resulting in DNA replication [2,3]. In turn, the E2F transcription factor initiates a positive feedback loop, promoting transcription of the type E-cyclins, which activate CDK2 and other proteins, with further phosphorylation of RB, thus resulting in DNA synthesis [4].

Other protein families also regulate and control cyclin D–CDK4/6 activity, such as cyclin-dependent kinase inhibitors (CKIs) [5]. Of note, the INK4 protein p16 can be induced by growth factor-β (TGFβ) signaling, and is able to bind to CDK4 and 6, decreasing cell progression from G1 phase to S phase, and therefore acting as a tumor suppressor, in opposition to the ER and CDK4/6 signaling stimuli [6,7].

Knowledge of this physiopathology enabled a plethora of trials exploring CDK4/6 inhibitors in monotherapy or in combination with antiendocrine agents in patients diagnosed with metastatic hormone receptor (HR)-positive BC, as well as ongoing clinical trials exploring anti-HER2 therapy in combination with CDK4/6 inhibitors, in HER2-positive disease. Indeed, in vitro studies conducted in estrogen receptor (ER)-positive cell lines, such as MCF-7 and T47-D, and also in HER2-positive BC cell lines, demonstrated that CDK4/6 inhibitors were capable of limiting proliferation and inducing cell cycle arrest in both cell lineages, providing a strong and robust rationale for CDK4/6 inhibitors’ development in clinical practice [8].

This review will focus on the recent efforts to more precisely characterize mechanisms of sensitivity and resistance to these drugs, both on the molecular and clinical characterization level.

## 2. Clinical Activity of CDK4/6 Inhibitors

Since the publication and approval of palbociclib based on the randomized phase 2 study PALOMA 1, extensive clinical data have demonstrated great activity achieved with the combination of CDK4/6 inhibitors plus endocrine therapy (ET). The PALOMA-1/TRIO-18 trial was an open-label randomized phase 2 study that enrolled postmenopausal women with metastatic HR-positive HER2-negative BC who had not received previous systemic treatment in the advanced disease setting. Patients were randomized to letrozol 2.5 mg daily, or the same ET plus oral palbociclib 125 mg for 3 weeks followed by 1 week off, in a 28-day duration cycle [9]. Researchers demonstrated a median progression-free survival (PFS) of 10.2 months for the letrozol group compared with 20.2 months for the combination of palbociclib plus letrozol (HR 0·488, 95% CI 0·319–0·748; *p* = 0·0004). The objective response rate (RR), clinical benefit rate (CBR), and median duration of response also favored combination therapy compared ET alone.

The phase 3 PALOMA 2 trial followed this study, mirroring and confirming its results [10]. Following roughly the same trial design, PALOMA 2 demonstrated a similar reduction in the risk of disease progression or death achieved with the combination of letrozol and palbociclib [10]. Of note, the median PFS was 24.8 months in the palbociclib letrozol group compared to 14.5 months in the placebo letrozol group (HR 0.58; 95% CI, 0.46 to 0.72; *p* < 0.001). In consequence, on March 2017, the U.S. Food and Drug Administration (FDA) granted regular approval to palbociclib for the treatment of postmenopausal metastatic HR-positive BC patients in combination with an aromatase inhibitor as the initial endocrine-based therapy. This trial was recently updated, and with a more mature follow-up of over 37 months, the median PFS benefit was sustained, namely 27.6 months for palbociclib plus letrozol versus 14.5 months for letrozol alone (HR 0.563; *p* < 0.000001). Furthermore, this benefit was consistent across all patient subgroups [11,12].

Palbociclib was also evaluated in the endocrine-resistant setting. The PALOMA 3 trial randomized patients that had progressed on previous endocrine therapy to fulvestrant plus palbociclib or fulvestrant plus placebo. The median PFS was 9.5 months in the fulvestrant plus palbociclib cohort compared to 4.6 months for patients allocated to fulvestrant alone (HR 0.46, 95% CI 0.36–0.59, *p* < 0.0001), setting palbociclib as a valuable option for patients in this scenario [13]. At the 2018 European Society for Medical Oncology (ESMO) conference, PALOMA-3 was update [14]. This presentation demonstrated the first mature OS analysis from a phase 3 study of a CDK4/6i in the treatment of HR-positive HER2-negative BC patients. Representing 44.8 months of follow up, and approximately 60% data maturity, the investigators demonstrated a non-statistically significant improvement of 6.9 months in OS for patients randomized to fulvestrant plus palbociclib, namely 34.9 months for combination therapy, compared to 28 months for fulvestrant alone (stratified HR 0.81, 95% CI 0.64–1.02, *p* < 0.042). A subgroup analysis demonstrated that the cohort of patients with previous sensitivity to endocrine therapy experience a more pronounced improvement in OS, with an absolute difference in median OS of 10 months. Additionally, with a more mature follow up, the median PFS was 11.2 months in the fulvestrant plus palbociclib cohort, compared to 4.6 months for patients allocated to fulvestrant alone (HR 0.49, 95% CI 0.39–0.62, *p* < 0.0001). Additionally, time from randomization to post progression chemotherapy also favored the group of patients who received palbociclib, being 17.6 months for combination therapy compared to 8.8 months for endocrine therapy alone.

Data on ribociclib, another CDK4/6 inhibitor, confirmed clinical activity from the targeted agent combined with the aromatase inhibitor letrozol in metastatic HR-positive postmenopausal women who had not received previous therapy in the metastatic setting [15]. Following this publication, MONALEESA 7 was an important trial designed specifically to evaluate CDK4/6 inhibitors in premenopausal patients, and demonstrated that these women also derived great benefit with the addition of ribociclib plus tamoxifen or a non-steroidal aromatase inhibitor combined with monthly goserelin [16]. In fact, the patients treated with ribociclib plus ET had a clinically meaningful 11-month improvement in median PFS compared to those receiving placebo plus ET, regardless of the endocrine therapy partner (tamoxifen or non-steroidal aromatase inhibitor), a similar benefit to that witnessed in the cohort of postmenopausal women. Data on overall survival demonstrated a significant reduction in the risk of death among patients who received ribociclib compared to placebo. The estimated overall survival at 42 months was 70.2% in the ribociclib group compared to 46.0% in the placebo cohort of patients (HR for death, 0.71; *p* < 0, 00973) [17].

MONALEESA-3 also provided another endocrine agent option to be combined with ribociclib in the first- and second-line disease setting. Importantly, this study included a mixed patient population cohort, recruiting patients both in the first- and second-line setting. In this study, the estrogen receptor antagonist that downregulates ER, fulvestrant, was combined with ribociclib. A consistent benefit in PFS was obtained with the combination therapy, both in patients with and without previous line of therapy [18,19]. At a median follow up of 39.4 months,  patients who received ribociclib experienced a statistically significant prolongation in overall survival compared to those who received placebo. The median OS was not reached for the CDK inhibitor cohort compared to 40.0 months for the placebo group (HR 0.724, *p* < 0.00455 [20]. Considering the excellent survival outcomes associated with the combination of fulvestrant and ribociclib in the first-line setting, the best endocrine partner to be combined with a CDK4/6 inhibitor was evaluated in the phase II PARSIFAL study [15]. A total of 486 patients diagnosed with advanced BC with no prior therapy in the advanced setting and endocrine-sensitive disease were randomized to receive letrozol or fulvestrant combined with palbociclib. At the median follow-up of 32 months, median PFS was 27.9 months with palbociclib and fulvestrant and 32.8 months with palbociclib and letrozol (HR: 1.1, *p* < 0.321). Additionally, there was no difference for both endocrine partners according to the patient characteristics, such as those without visceral involvement or in those with de novo or recurrent metastatic disease.

Finally, a third agent targeting CDK4/6, abemaciclib, was also extensively evaluated in metastatic HR-positive BC. The MONARCH 3 phase 3 randomized trial evaluated postmenopausal women with HR-positive advanced BC who had no prior systemic therapy in the advanced setting. Patients received abemaciclib or placebo plus a non-steroidal aromatase inhibitor. Similar to the experience from palbociclib and ribociclib, the addition of abemaciclib resulted in a reduced risk of progression or death by approximately 50% (HR 0.54; 95% CI, 0.41 to 0.72; *p* = 0.000002 [21]. Among patients in the second-line setting, the MONARCH 2 study evaluated abemaciclib combined with fulvestrant, and demonstrated that the combination significantly improved the PFS and overall response rate compared to fulvestrant alone, also consolidating this agent as an option in patients resistant or refractory to an aromatase inhibitor [22]. Data on overall survival were also presented for the MONARCH 2 study. Patients randomized to abemaciclib experienced a statistically significant 10-month improvement in overall survival compared to patients who received fulvestrant alone. In detail, the median OS was 46.7 months for abemaciclib plus fulvestrant and 37.3 months for placebo plus fulvestrant (HR 0.757; 95% CI 0.60 to 0.94; *p* = 0.01) [23].

In distinction from other CDK4/6-blocking drugs, abemaciclib was evaluated as a single agent in the phase 2 MONARCH 1 study. Abemaciclib 200 mg was administered as monotherapy in a continuous schedule every 12 h until disease progression or unacceptable toxicity. In a heavily previously treated metastatic BC cohort, with approximately 90% of patients having visceral disease and 50% harboring three or more metastatic disease sites, abemaciclib was capable of inducing a primary objective confirmed response rate of 19.7%, clinical benefit rate of 42.4%, and median PFS of 6.0 months [24]. The next MONARCH trial also evaluated abemaciclib in monotherapy in HR+ HER2-negative advanced BC who had progressed on or after prior endocrine therapy, and had previously received chemotherapy. Patients were randomized to abemaciclib 150 mg twice daily plus tamoxifen 20 mg, abemaciclib 150 mg twice daily alone, or abemaciclib 200 mg twice daily. The study confirmed the single-agent activity of abemaciclib in heavily pretreated patients. The efficacy of abemaciclib monotherapy at 150mg was similar to 200 mg [25].

Abemaciclib has the potential to achieve responses in the central nervous system. A recent multicenter open-label phase 2 trial evaluated the safety and efficacy of abemaciclib in patients with HR+ HER2-negative leptomeningeal disease. The median OS in patients who received abemaciclib in monotherapy was 8.4 months, which is favorable compared with historical data [26]. An updated analysis of this study in a cohort of hormone receptor-positive breast cancer patients was recently presented. The study primary endpoint was the objective intracranial response rate (OIRR) according to the neuro-oncology brain metastasis response assessment criteria. Patients recruited to this study were heavily pretreated, with a median of four prior lines of therapy. The OIRR was 6% and intracranial clinical benefit rate (complete or partial response, stable disease persisting for ≥ 6 months) was 25%, demonstrating a modest activity of abemaciclib as monotherapy.

Additionally, more data is accumulating in the sense that patient-reported outcomes (PROS) were improved in patients who received combination therapy with CDK4/6i compared to endocrine therapy alone. A recent report demonstrated that there was a numerical trend favoring ribociclib versus placebo for time to deterioration in global health-related quality of life (HRQoL). Additionally, HRQoL was improved or maintained compared to baseline during treatment but worsened when treatment was stopped in both arms, suggesting that disease progression is a major responsive for HRQoL deterioration [27].

Similarly, among premenopausal patients, investigators from MONALEESA-7 also demonstrated that ribociclib combined with endocrine therapy resulted in a delayed time to HRQoL deterioration. Among important symptoms related to disease, pain and fatigue were improved with combination therapy. Besides, the delayed disease progression experienced with ribociclib was associated with improved HRQoL [28].

Additionally, CDK4/6i inhibitors are being evaluated in patients with HER2-positive disease. The PATRICIA study is a prospective open-label multicenter phase II trial that evaluated the combination of palbociclib with trastuzumab in HER2-positive BC patients, with either HR-positive or -negative disease. The clinical benefit rate was 73% in luminal versus 31% in non-luminal patients (*p* = 0.031) [29]. The MONARCHER study confirmed the activity of CDK4/6i in patients with hormone receptor-positive HER2-positive BC. The study randomized 237 women with advanced BC who had been previously treated with at least two prior HER2-directed therapies. Patients were randomized 1:1:1 to receive abemaciclib combined with trastuzumab and fulvestrant (Arm A) versus abemaciclib plus trastuzumab (arm B), or trastuzumab plus the investigator’s choice of chemotherapy (arm C). Patients assigned to receive abemaciclib combined with trastuzumab and fulvestrant had a significant improved PFS compared to the other two arms. This group experienced a median PFS of 8.3 months, compared to 5.7 months for arm C (HR 0.673; *p* < 0.05). For arm B, the median progression-free survival was 5.6 months, comparable to arm C (HR 0.943; *p* < 0.77 for arm B vs. arm C). The objective response rate also favored the triplet combination with abemaciclib [30].

**Figure 1:**
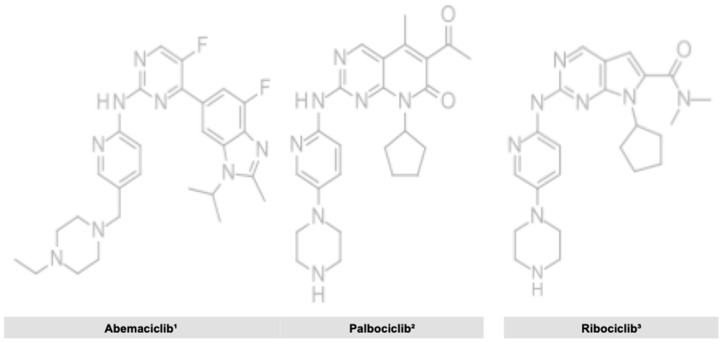
Chemical structure of the different CDK inhibitors. **1**. Abemaciclib [31]. **2**. Palbociclib [32]. **3**. Ribociclib [33].

Figure 1 displays the chemical structure of the three different CDK inhibitors.

A recent meta-analysis demonstrated that CDK4/6i therapy added to endocrine therapy in patients with advanced HR+ HER2-negative BC substantially improved PFS (HR 0.54, *p* < 0.00001) and OS (HR 0.77, *p* < 0.00001), irrespective of the endocrine partner, line of therapy, or menopausal status, corroborating individual clinical trial data [34].

In summary, palbociclib, ribociclib, and abemaciclib in combination with endocrine therapy significantly improved the PFS overall response rate, and overall survival compared to placebo plus endocrine therapy in patients with metastatic HR-positive HER2-negative BC, both in first- and second-line disease setting. Table 1 and Table 2 lists the main trials that evaluated these different CDK4/6 inhibitors in first and later lines of therapy. Currently, the FDA has approved all three drugs in women with metastatic HR-positive HER2-negative BC in distinct advanced disease settings. Palbociclib and abemaciclib are approved in combination with an aromatase inhibitor as initial therapy, or with fulvestrant in women with disease progression following endocrine therapy. Ribociclib is approved in the first-line setting combined with an aromatase inhibitor. Of note, abemaciclib is the only agent approved as monotherapy for the treatment of advanced BC patients with disease progression following endocrine therapy and prior chemotherapy.

## 3. Toxicity and Financial Impact

Undeniably, these drugs have great clinical utility and add value in the therapy armamentarium for patients with HR-positive metastatic BC. Nevertheless, toxicity is considerably increased, compared to endocrine therapy alone. Among patients who received abemaciclib, diarrhea, neutropenia, fatigue, and nausea are the most common side effects observed. Diarrhea is more commonly observed upon therapy with abemaciclib, presents early in treatment, is typically low grade, and rarely leads to dose modifications. In MONARCH 2, for example, approximately 85% of patients experienced diarrhea, although diarrhea grade 3 occurred in 13.4%, with no occurrence of grade 4 events [16]. On the basis of laboratory alterations, the most common abnormalities were decreased neutrophils and hemoglobin levels, and an increased serum creatinine level. Although 46% of patients experienced a decreased neutrophil count, neutropenia grade 3 or 4 occurred in 26.5% and febrile neutropenia is a rare complication.

As for palbociclib and ribociclib, the most common grade 3 or 4 adverse events are neutropenia and leucopenia. Across trials, rates of neutropenia grade 3 and 4 occur in approximately 50–60% of patients, although febrile neutropenia is extremely rare, usually in the order of 2% to 3%. In addition to hematological toxicity, ribociclib can lead to alterations in electrocardiogram, with prolongation of the QTcF interval. In MONALEESA 7, an increase of more than 60 ms from baseline in the QTcF interval occurred in 10% of individuals taking ribociclib, and dose interruptions or reductions due to QTcF interval prolongation occurred in 4% [16]. The combination of ribociclib and tamoxifen was associated with an incidence of 16% of QTcF prolongation, compared to 7% for patients receiving a non-steroidal aromatase inhibitor. Fortunately, no cases of the Torsades de Pointes, a potentially life-threatening arrhythmia that can lead to sudden cardiac death, have occurred. Ribociclib can also lead to an increase in alanine and aspartase amino-transferase. In fact, the most common reasons for interruption of any component of study treatment owing to adverse events suspected to be related to study treatment were alterations in liver enzymes [16].

Besides the hematologic toxicity, fatigue, nausea, and arthralgia are the most common adverse events with palbociclib and ribociclib. Patients must also be aware of the risk of alopecia with these agents. A recent meta-analysis found a relative risk for all-grade alopecia with the addition of CDK4/6 inhibitors to endocrine therapy of 2.14 (95% CI: 1.23–3.73, *p* < 0.007) [35]. Although most events are defined as low grade, patients may experience significant distress upon manifestation of hair loss, possibly impacting their quality of life.

In addition to increased clinical toxicity, the incorporation of CDK4/6 inhibitors to endocrine therapy entails a considerable financial augmentation in cost. In parallel to the ability of more efficiently tackling tumors, a dilemma in private health insurance and government agencies worldwide is the real struggle to deliver these drugs. Additionally, patients’ ability to co-pay the medication can limit access to health care, and represent a major barrier to access.

Identifying patients most likely to benefit from the addition of CDK4/6 inhibition to endocrine therapy is extremely important, and may potentially spare individuals from the toxicity encountered with these medications, as well as more rationally employ financial resources.

Additionally, the timing of CDK4/6 inhibitors’ incorporation in the treatment strategy of patients with HR-positive metastatic BC is still a matter of debate. The literature is not clear regarding which individual can be treated exclusively with an aromatase inhibitor upfront, followed by CDK4/6 inhibition with fulvestrant in the second line as a treatment strategy.

Currently, PALOMA 1, MONARCH-2, PALOMA-3, and MONALEESA 3 have already reported overall survival (OS) results. In PALOMA-1, OS was only numerically improved, from 37.5 months for the palbociclib plus letrozol group to 34.5 months for letrozol alone (HR = 0.897 (95% CI: 0.623, 1.294); *p* = 0.281) [17,36]. However, it should be taken into account that this phase II study was not designed for overall survival improvement, and the long post progression survival curves might make it difficult to observe statistically significant differences. In PALOMA-3, as previously described, OS was numerically improved, resulting in an absolute difference of 6.9 months, favoring the CDK4/6i plus endocrine therapy group [14]. Of note, patients with previous endocrine therapy sensitivity experienced a 10-month statistically significant improvement in OS. Contrastingly, in a patient population who also had progressed on previous endocrine therapy, subgroup analysis from MONARCH-2 revealed that those individuals with primary endocrine resistance derived the greatest benefit from CDK4/6 inhibitor addition to fulvestrant. In alignment, similar divergent results have been observed for PFS in these subgroups in PALOMA-3 and MONARCH 2. The mechanism behind these differences is currently unknown. Additional studies are warranted to prospectively clarify these discrepancies.

Therefore, both clinical and molecular markers to identify groups most likely to benefit from CDK4/6 inhibitors are of great interest. Subgroup analysis from the PALOMA, MONALEESA, and MONARCH trial series were not able to clinically identify a potential patient characteristic that did not derive benefit from the addition of CDK4/6 inhibitors to endocrine therapy. In this review, we will describe the literature available on potential biomarkers of sensitivity/resistance to these drugs.

## 4. Patient Clinical Characteristics and Benefit from CDK4/6 Inhibitors

Efforts concentrated to identify clinical characteristics that could potentially lead to the characterization of a group of patients who could derive greater benefit from CDK4/6 inhibitors were made across all phase 3 randomized trials, including the PALOMA, MONALEESA, and MONARCH series of trials. Overall, researchers could not identify a group of patients that did not benefit from the addition of CDK4/6 inhibitors to endocrine therapy. Certain clinical characteristics were able to translate in a lesser absolute benefit from the addition of these targeted agents. Although some cohorts of patients, such as those with a prolonged treatment-free interval, bone-only metastasis, and absence of liver disease, may experience a comparatively better prognosis with endocrine monotherapy alone, the addition of a CDK4/6 inhibitor to endocrine therapy also resulted in a statistically significant prolongation of PFS and improved response rates.

Investigators from the PALOMA-2 and PALOMA-3 trials sought to evaluate the efficacy and safety of palbociclib plus endocrine therapy in patients with or without visceral metastases [18,37]. Overall, patients with prior resistance to endocrine therapy and visceral metastases experienced a median PFS that was substantially shorter compared to patients with no visceral metastasis. Of note, this group presented with a median PFS of 9.2 months with palbociclib plus fulvestrant versus 3.4 months with placebo plus fulvestrant (hazard ratio, 0.47; 95% CI 0.35–0.61), and an objective response rate (ORR) of 28.0% versus 6.7%, respectively. In contrast, among patients with non-visceral metastases, the median PFS was 16.6 versus 7.3 months (HR 0.53; 95% CI 0.36–0.77). An important observation from this analysis lies in the fact that even in the cohort of patients with poor clinical characteristics, mainly those with prior resistance to endocrine therapy and visceral disease, the addition of palbociclib to endocrine therapy was capable of delaying the deterioration of quality of life, reinforcing the importance of these drugs, even without an absolute increase in median months of PFS similar to the one observed in the cohorts of patients with more favorable clinical characteristics.

An exploratory analysis from over 1000 patients treated in the MONARCH 2 and MONARCH 3 trials demonstrated that the addition of abemaciclib to endocrine therapy led to an improvement in median PFS compared to endocrine therapy alone, with hazard ratios that ranged from approximately 0.3 to 0.5, regardless of clinical characteristics, such as the Eastern Cooperative Oncology Group performance status, tumor grade, progesterone receptor status, liver metastases, or bone-only metastasis [38]. This analysis also demonstrated that patients with a shorter disease-free interval from prior endocrine therapy appeared to have a poorer prognosis, and derived more benefit from the addition of abemaciclib compared to patients with a longer disease-free interval.

Another analysis was conducted in patients with liver metastasis at baseline across the MONARCH 1, 2, and 3 studies. Benefit from the addition of abemaciclib to endocrine therapy in this particular group, which historically only achieved a modest benefit from single-agent endocrine therapy, was observed. Additionally, abemaciclib monotherapy in the MONARCH 1 trial led to an ORR of 21.5% and a median PFS of 5.56 months in patients with liver disease, previously heavily treated [39]. Table 3 lists the specific drug activity in different clinical scenarios in the PALOMA2 and MONARCH 3 trials.

The benefit of CDK4/6 inhibitors in individuals with old age was also investigated. Pooled data from prospective randomized trials of abemaciclib, ribociclib, and palbociclib in combination with an aromatase inhibitor for the initial treatment of HR+ HER-negative BC patients were evaluated. This cohort of patients consisted of 1334 patients, with 42% being older than 65 years of age, and 24% 70 years or older [40]. Of note, both age groups derived benefit from the incorporation of CDK4/6 inhibitors into therapy, similarly to that observed in younger age groups. Nevertheless, older patients were more likely than their younger counterparts to discontinue treatment due to side effects. In this analysis, 20% of the patients 70 years of age or older discontinued treatment compared with 17% in the group older than 65 years, and 8% for those younger than age 65. It is important to emphasize that patients enrolled in clinical trials frequently have fewer comorbidities and less frailty, so the rates observed in the real world population might be even higher.

The presence of brain metastasis is associated with poor prognosis, even in the HR-positive HER-negative BC subtype [41]. Although the incidence of brain metastasis in this BC subtype is not as high as that experienced by the more aggressive subtypes, such as triple-negative and HER-2-positive BC, there was an interest in evaluating the activity of CDK4/6 inhibitors in central nervous system (CNS) metastasis [42]. Abemaciclib has previously demonstrated the capability to cross the blood–brain barrier, and prolong survival in an intracranial glioblastoma xenograft model [43]. In a recent study, abemaciclib was associated with a confirmed and durable response in CNS lesions of HR+ HER2-negative BC patients, with a clinical benefit rate of 17.4% [44]. Further investigation of this agent in patients with brain metastasis is ongoing.

In addition, a recent pooled analysis evaluating five registration trials for CDK4/6 inhibitors led by the FDA concluded that all patients benefited from the addition of CDK4/6 inhibitor to endocrine therapy, including patients with de novo metastatic disease, bone-only metastasis, or patients whose tumors were negative for progesterone receptor in immunohistochemistry [45].

In summary, based exclusively on clinical characteristics, one cannot identify a subgroup of the HR+ HER2-negative advanced BC patient subgroup that does not derive benefit from the addition of a CDK4/6 inhibitor to endocrine therapy, both in the resistance and sensitivity hormone setting. Therefore, the decision to incorporate these targeted agents in the front- or later-line setting is still a matter of debate. The SONIA trial (NCT03425838) is an investigator-initiated multicenter, randomized phase III study that evaluated whether the sequence of an aromatase inhibitor plus CDK 4/6 inhibitor in the first-line setting followed by fulvestrant in the second line is superior to the sequence of an aromatase inhibitor in the first line followed by the combination of fulvestrant plus CDK4/6 inhibitor in the second line in patients with advanced HR+ HER-negative BC previously untreated with systemic therapy for loco-regional recurrent or metastatic disease.

## 5. Molecular Biomarker Analysis Correlated with Resistance or Benefit from CDK4/6 Inhibitors

Based on the biologic interaction between the RB tumor suppressor and CDK4/6 previously described in this review paper, it is hypothesized that an intact RB tumor suppressor downstream of CDK4/6 is necessary for this class of agents to have an effect. In fact, in vitro negative RB tumor cell lines are resistance to CDK4/6 blockage [41]. Investigators from the PALOMA 3 trial evaluated driver mutations in patients receiving either placebo or a CDK4/6 inhibitor. Circulating tumor DNA (ctDNA) in 193 pairs of baseline and end of treatment (EOT) plasma samples from this study were evaluated. Additionally, some paired samples with high tumor purity were subjected to whol-exome ctDNA sequencing. RB1 mutations emerged at the end of therapy, upon progression, in 4.8% of patients receiving palbociclib plus fulvestrant. Of interest, no RB1 mutations emerged in the group who received fulvestrant alone, suggesting that this might represent a mechanism for therapy resistance, although the fact that only a small minority of patients receiving CDK4/6 inhibitor indeed developed RB mutation is not capable to fully address and explain resistance mechanisms to these drugs [46,47]. In fact, previous work demonstrated that many resistant cell lines to CDK4/6 inhibitors do not display RB loss or mutation [9].

A previous study, designed in an ex vivo model of breast tumor tissue, demonstrated that the models harboring loss of RB were not able to achieve a response to palbociclib. In brief, investigators determined the cytostatic response to the CDK4/6 inhibition through suppression of the proliferation marker KI67. It is important to emphasize that there was a strong positive correlation between the Ki67 marker of the primary tumor, and the corresponding explanted tumor tissue. Upon exposure to palbociclib, most ex models achieved suppression in proliferation. Contrasting with these findings, all models that failed to achieve suppression in proliferation upon treatment with palbociclib harbored RB tumor suppressor loss [48].

A joint analysis from PALOMA-2 and PALOMA-3 studies that included patients with bone-only and non-bone-only metastasis evaluated tissue for mRNA profiling through the EdgeSeq Oncology Biomarker Panel. Bone-only metastasis patients displayed a higher incidence of luminal A disease, and a lower rate of luminal B disease. In both studies, baseline ESR1, cyclin E, and CDK 4 gene expression levels were comparable between bone-only and non-bone-only patients. An elevated CDK4 gene expression level was associated with resistance to single-agent letrozol in PALOMA-2, while lower cyclin E gene expression predicted for the palbociclib effect in PALOMA-3 [49].

Furthermore, investigators from the MONALEESA-3 trial extensively evaluated a variety of biomarkers in order to identify those that could predict benefit/resistance to ribociclib. At ESMO 2018, Neven et al. demonstrated that total Rb, p16 protein expression, CCND1, CDKN2A, and ESR1 messenger RNA (mRNA) levels could not identify patients who did not derive benefit from CDK4/6 inhibition [50]. The same group of investigators evaluated baseline ctDNA from plasma samples of 692 patients, analyzed using next-generation sequencing with a targeted panel of approximately 550 genes. Progression-free survival was consistently improved with the addition of ribociclib to endocrine therapy, regardless of genomic alteration, including those harboring alterations in genes, such as PIK3CA, ESR1, TP53, CDH1, FGFR, or cell cycle-related and the mitogen-activated protein kinase (MAPK) pathway genes [51]. Lastly, gene expression in baseline tumor samples from 531 patients using tumor samples collected before treatment using the NanoString 800-gene nCounter^®^ GX Customized Panel from the MONALEESA-3 trial was presented at the San Antonio Breast Cancer Conference in 2019. The PFS benefit observed with the addition of ribociclib to therapy was observed across all gene expression subgroups [52].

Recently, investigators from the MONALEESA trials compiled data from 1503 patients recruited in the MONALEESA 2, 3, and 7 trials. In this analysis, baseline ctDNA was evaluated through next-generation sequencing, using a targeted panel of 557 genes. Patients with alterations in FRS2, PRKCA, MDM2, ERBB2, AKT1, and BRCA1/2 had a trend towards an increased PFS benefit for treatment with ribociclib compared to placebo. In contrast, individuals with alterations in CHD4, BCL11B, ATM, or CDKN2A/2B/2C derived little to no added PFS benefit when treated with ribociclib compared to placebo [53].

The expression of RB1, CCND1 (cyclin D1), and CDKN2A (p16) has been associated with suggested resistance to CDK4/6 inhibitors [21,54]. In vitro, higher levels of RB1 and CCND1, and lower levels of CDKN2A were linked to sensitivity to therapy [55]. In order to clinically evaluate those findings, investigators from the PALOMA 1 grouped patients recruited in this trial into two distinct groups [10]. Group 1 included patients enrolled on the basis of their hormone receptor status alone (HR positive and HER2 negative) and group 2 included patients that had cancers with amplification of CCND1, loss of p16, or both. Among patients in group 2, the median PFS was 18.1 months for the combination of palbociclib and letrozol and 11.1 months for letrozol alone (HR 0·508, 95% CI 0.303–0.853; *p* = 0.0046). This benefit compared similarly for the cohort of patients in group 1. Therefore, evaluation of alterations in cyclin D1 and p16 were not predictive of palbociclib benefit in this phase I clinical trial.

Another potential mechanism of acquired resistance to CDK4/6 inhibition is increased CDK6 expression in ER-positive BC cells treated with palbociclib. Increased expression of CDK6 was implicated in resistance to CDK inhibitors in KRAS-mutant NSCLC cell lines that were initially sensitive to palbociclib [22,56]. In a model of an MCF-7-resistant cell line with exposure to the CDK4/6 inhibitor LY5219, the investigators observed a 7-fold increase in CDK6 mRNA levels in the resistant cells, with concomitant increases in CDK6 protein levels, as compared with sensitive cells. This finding is consistent with an acquired amplification in the drug target, CDK6 [57], suggesting that long-term exposure of the models to the CDK inhibitor led to multiple independent clones that had evidence for amplification of the CDK6 kinase. Corroborating the role of CDK6 expression in determining resistance to CDK4/6 inhibitors, investigators suppressed CDK6 levels in these resistant cell lines using short-hairpin RNAs against CDK6. Pursuant, this suppression was capable of restoring the sensitivity of resistant cells to LY5219, a selective inhibitor of CDK4/6. Furthermore, in this study, two other models were associated with resistance to CDK4/6 inhibitors, including RB mutation and cyclin E1 overexpression [57].

CDK6 expression may also be induced by a loss of FAT1 tumor suppressor. FAT1 can regulate CDK 6 expression through the Hippo signaling pathway, as this pathway can result in the accumulation of YAP and TAZ transcription factors on the CDK6 promoter [58].

Additionally, CDK4 amplification and overexpression might be associated with diminished sensitivity to CDK inhibitors. For example, in a rhabdomyosarcoma cell line and xenograft model, there was diminished sensitivity to CDK4/6 inhibition associated with CDK4 amplification and overexpression [59].

In order to investigate mechanisms of resistance to CDK inhibitors, researchers performed a library of 559 sequence-validated kinase open reading frame clones in estrogen receptor-positive MCF-7 cells treated with fulvestrant plus or minus ribociclib. The authors found that FGFR1 overexpression induced less sensitivity to the CDK inhibitor plus endocrine therapy. Furthermore, PDX models from patient-derived FGFR1-amplified ER+ tumors responded to the triple combination of an FGFR1, Erα, and CDK4/6 inhibitor [60].

Molecular biomarker analysis was also performed in patients enrolled in the MONALEESA-2 trial. Here, tumor samples were evaluated for gene expression using the NanoString 230-gene nCounter^®^ GX Human Cancer Reference panel. Correlations between the gene expression level and PFS were accessed, and patients were categorized into low and high messenger RNA expression groups, using a 10% cut-off for RB1 and the median expression as the cut-off for other genes [61]. No biomarker could identify a subgroup of patients that derived no benefit from the addition of ribociclib to letrozol, including high versus low expression of ESR1, E2F, CDK2, CCNE1, CDK2, and FGFR1. Additionally, genes implicated in alternative pathways in breast cancer, such as the PI3K and MAPK pathways, could not predict ribociclib benefit.

Additionally, investigators from the MONARCH 3 trial tried to correlate genomic alterations detected in baseline ctDNA with clinical outcomes, such as PFS and the objective response rate. Baseline ctDNA results were evaluated for 295 patients, with the great majority, namely 83%, harboring one or more detectable genomic alterations. The authors found that genomic alterations were associated with a shorter PFS in patients allocated to placebo plus endocrine therapy. Importantly, abemaciclib improved outcomes for all genomically identified subgroups, including those with FGFR alterations, for example [62].

Until recently, as previously described, no molecular alterations correlated with a lack of benefit from CDK4/6 inhibitors in clinical practice, among the totality of trials that evaluated CDK4/6 inhibitors in patients with HR-positive HER2-negative breast cancer. Nevertheless, unprecedented research identified CCNE1 (cyclin E1) expression as a potential palbociclib resistance marker. In this analysis, 302 patients who participated in the PALOMA 3 trial had tumor tissue analyzed by mRNA profiling. Benefit from palbociclib was greater in patients with low tumor CCNE1 expression. Overall, in patients with low CCNE1 expression, the median PFS with palbociclib plus fulvestrant was 14.1 months compared to 4.8 months for placebo and fulvestrant (hazard ratio 0.32). In contrast, among patients with high tumor CCNE1 expression, the median PFS with palbociclib plus fulvestrant was 7.6 months versus 4.0 months for placebo and fulvestrant (hazard ratio 0.85; interaction *p* = 0.0024). Additionally, expression levels of CDK4, CDK6, cyclin D1, and RB1 were not associated with benefit from palbociclib, including patients with either luminal A or luminal B subtypes [63]. Additionally, in this analysis, high E2F expression predicted relative resistance to palbociclib, corroborating the hypotheses of increased levels of E2F expression being implicated in resistance to CDK4/6 inhibitors generated in previous in vitro studies.

Other mutations in genes studied in BC were also previously analyzed in cohorts of patients being treated with CDK inhibitors. ESR1 mutations, which occur rarely in primary BC but have increased in prevalence in patients treated with aromatase inhibitors [64,65], were evaluated in patients enrolled in the PALOMA-3 trial. In this analysis, ESR1 mutations were found in the plasma of 25.3% of patients, of whom 28.6% were polyclonal, with mutations associated with acquired resistance to prior AI. Fulvestrant plus palbociclib was associated with improved PFS compared with fulvestrant plus placebo, regardless of ESR1 status, with the hazard ratio for PFS for the mutant being 0.43 (95% CI, 0.25 to 0.74; *p* < 0.002) and for ESR1 wild-type patients 0.49 (95% CI, 0.35 to 0.70; *p* < 0.001) [66]. Treatment with CDK4/6 inhibitors does not prevent selection of ESR1 mutations in later lines of therapy, with mutations being enriched during therapy, compared to the baseline of patients receiving palbociclib and letrozol therapy [67].

Genomic profiling of hormone receptor-positive metastatic breast cancer, with characterization of acquired genomic alterations was recently evaluated. Matched sequencing of samples pre and post-treatment with CDK4/6i were prospectively assessed. Scientists found multiple genes to be specifically enriched in the post-CDK4/6i samples, such as alterations in RB1, in effectors of PI3K/AKT signaling (excluding PIK3CA), cell cycle (CDKN2A loss), and Hippo signaling [68].

Therefore, although in vitro and ex vivo experiment models suggested potential biomarkers for the identification of mechanisms of resistance to CDK4/6 inhibitors, molecular evaluation of the expression from a variety of genes, such as ESR1, E2F, CDK2, CCNE1, CDK2, FGFR1, CCND1, and CDKN2A, among patients recruited in clinical trials failed to detect a biomarker that could select patients who do not derive benefit from these drugs. Researchers face an important challenge, since the great majority of tumor tissues analyzed in clinical trials are indeed from primary breast tumor samples, instead of the actual progressive metastatic disease site. The recognition of molecular alterations and evolution found in metastatic lesions, compared to the primary tumor, is well recognized [69]. Those genomic alterations, which frequently founder under treatment-selective pressure, can induce a complete different molecular portrait of alterations, including distinct somatic mutations, copy number variations, and gene fusions, that can ultimately promote a different phenotype and behavior in various cell processes.

## 6. Detecting Dynamic Circulating Tumor DNA Alterations and Correlating with Response and Resistance to CDK Inhibitors

Although not incorporated and formally recommended in clinical practice by ASCO guidelines [70], it is well established that levels of ctDNA fluctuate during cancer treatment. The levels can vary according to disease response to therapy, in a diversity of cancer types, offering the potential for non-invasive monitoring of disease [71,72]. Additionally, peripheral blood genomic evaluation has the potential to access tumor heterogeneity from multiple metastatic sites, truly representing the relevant mutations that might be driving tumor progression in a certain moment of disease evolution. [73,74]. Variations in ctDNA could potentially be used to predict treatment response, sensitivity, and resistance, allowing for treatment tailoring. In an analysis evaluating the predictive role of longitudinal ctDNA assessment in the PALOMA-3 study, researchers demonstrated that early circulating tumor DNA dynamics according to the rate of PIK3CA mutations predicted sensitivity to palbociclib among individuals treated in the PALOMA-3 trial [75]. This data suggested that cancers with incomplete cell cycle arrest upon treatment with palbociclib might continue proliferation while on treatment, consequently leading to the release of tumor DNA in the circulation.

Of interest, PIK3CA mutation detected at baseline was not a predictive biomarker for palbociclib plus fulvestrant benefit. Nevertheless, dynamic alterations might provide further information. Researchers defined a “circulating DNA ratio”, comparing the ratio of mutant copies per mL on treatment at day 15 after palbociclib initiation relative to the ratio found at baseline. Of note, patients randomized to palbociclib plus fulvestrant had a lower PIK3CA circulating DNA ratio compared to patients allocated to fulvestrant plus placebo, suggesting an important role of ctDNA turnover to predict response to targeted therapies [75].

Furthermore, the same study showed that the dynamics of ESR1 mutations is commonly sub clonal, and did not predict sensitivity to CDK4/6 inhibition. In reality, ESR1 mutant ctDNA was markedly decreased in both the combination of palbociclib and fulvestrant and also among patients receiving fulvestrant alone. However, this does not associate with long-term improvement in survival outcomes on fulvestrant alone relative to patients with wild-type ESR1 [75]. Recently, investigators reported the first results from the PADA-1 trial, in which the utility of monitoring the onset of ESR1 mutation in cell-free DNA from patients receiving first-line aromatase inhibitor combined with palbociclib is being evaluated [76]. Among the 1017 patients, 3.2% had a detectable circulating ESR1mut at inclusion. Upon treatment with an aromatase inhibitor and palbociclib, 78% of these patients had a clearance in ESR1 mutation, and achieved a median PFS of 17.5 months, suggesting activity of palbociclib in this subset of patients. Remarkably, patients with ESR1 mutation had a shorter PFS compared to those without ESR1 mutation, with an estimated HR of 2.8.

Another technique of interest is the extraction of exosomes from RNA derived from plasma. In a previous study that prospectively enrolled 34 metastatic BC patients, the comparison of mRNA levels was able to distinguish patients who benefited from CDK4/6i treatment from those who presented with progressive disease at initial evaluation. Plasma was collected at baseline and at the first evaluation for the expression of thymidine kinase 1 (TK1), and CDK 4, 6, and 9 by digital droplet PCR in patients receiving a non-steroidal aromatase inhibitor plus palbociclib. The comparison of changes in the mRNA expression between TK1, CDK 4, 6, and 9 at baseline compared to the first evaluation was statistically significant for TK1 (partial response (PR) +stable disease (SD) versus progressive disease (PD) *p* < 0.009), CDK4 (PR+SD versus PD *p* 0.020), CDK6 (PR+SD versus PD *p* < 0.047), and CDK9 (PR+SD versus PD *p* < 0.008) [77].

Data on these dynamic alterations in ctDNA observed through treatment with CDK4/6 inhibitors could be a promising biomarker in a scenario in which no clinical characteristic or molecular abnormality can reliably identify a group of individuals who does not derive benefit from these drugs. Although the ideal time point for comparative assessment is unknown, and the degree of uncertainty concerning the true truncal or sub clonal status of the PIK3CA is a major challenge, such study would foment clinical trials testing the hypothesis that a change in treatment strategy based on early ctDNA dynamics may improve outcome and safe costs, by switching to another modality or adding additional treatment for patients with inadequate ctDNA suppression.

## 7. Is There A Distinct Resistance Mechanism to Each Specific CDK4/6 Inhibitor?

Clinically, albeit there are minor differences in survival outcomes of distinct patient subgroups’ profiles among the three different approved CDK inhibitors, there is no clear major difference in clinical practice between palbociclib, ribociclib, and abemaciclib. Recently, researchers interrogated if resistance mechanisms were homogenous for all approved drugs, generating in vitro models to examine mechanisms of resistance to different CDK4/6 inhibitors. In brief, MCF7 and T47D Palbociclib-resistant and MCF7 abemaciclib-resistant cells’ organoids derived from patient-derived xenografts were elected for investigation. Western blot analysis revealed dose-dependent downregulation of ERα, Rb, p-Rb, and p27, while levels of cyclin E and p-CDK2 increased in a stepwise fashion in palbociclib-resistant cells, which were only partially cross-resistant to abemaciclib, suggesting different resistance mechanisms to both drugs. Of note, RAD51D, crucial for homologous recombination, was downregulated only in abemaciclib-resistant cells, suggesting a potential role for drugs that target this pathway, such as poly ADP ribose polymerase (PARP) inhibitors, to circumvent abemaciclib resistance [78].

Other group investigated mechanisms of resistance between abemaciclib and ribociclib, employing resistant cell lines to each drug. Of interest, ribociclib-resistant cell lines demonstrated low sensitivity to abemaciclib and vice versa, implying an acquired cross-resistance. Additionally, CDK6 levels were upregulated in abemaciclib-resistant models but remained unaltered in ribociclib resistance, suggesting that the mechanism of resistance between ribociclib and abemaciclib might be different [31].

## 8. Conclusions

The addition of CDK4/6 inhibitors to endocrine therapy, either in the first-line setting or after progression to an aromatase inhibitor, significantly improved progression-free and overall survival compared to endocrine therapy alone in the treatment of post- and premenopausal women with advanced HR-positive HER2-negative breast cancer. The benefit is consistent, regardless of the number of prior treatments received, menopausal status, age, ductal or lobular histology, progesterone receptor status, and metastatic disease sites.

Currently, CDK4/6 inhibitors are incorporated and established as a new standard treatment for advanced HR-positive HER2-negative breast cancer. A greater delay to cytotoxic chemotherapy exposure, and quality of life scores’ preservation are among the most important endpoints in a group of patients with incurable disease, in which physical, emotional, and functional wellbeing are extremely important.

Despite the unequivocal improvement in a variety of survival endpoints achieved with the CDK4/6i combination with endocrine therapy, resistance ultimately occurs. Better knowledge of these mechanisms may lead to triplet combinations with other targeted therapies, which may prevent or delay treatment resistance. Furthermore, combinations of CDK4/6i with other agents, such as immune checkpoint inhibitors, are also being explored. Another important unanswered question is the value of maintaining CDK4/6 inhibition beyond progression.

Until the present day, no molecular or clinical biomarker is able to reliably identify a group of patients who do not derive benefit from the addition of these drugs to endocrine therapy. Further research is needed to differentiate subgroups of patients, based on molecular biomarkers, who will benefit from the addition of CDK4/6 to therapy. Hopefully, patients will be able to receive a more personalized treatment, which will spare them from drugs that add toxicity and financial cost, concentrating the treatment combination for the individuals who will definitively achieve a benefit.

## Figures and Tables

**Table 1:** CDK4/6 inhibitors in the first-line setting.

Clinical Trial	Endocrine Agent	CDK4/6 Inhibitor	PFS	HR
PALOMA 1	Letrozol (L)	Palbociclib (P)	10.2 months (L) 20.2 months (L + P)	HR = 0·488, 95% CI = 0·319–0·748, *p* = 0.0004
PALOMA 2	Letrozol (L)	Palbociclib (P)	14.5 months (L) 24.8 months (L + P)	HR = 0.58; 95% CI = 0.46 to 0.72; *p* < 0.001
MONALEESA 2	Letrozol(L)	Ribociclib (R)	14.7 months (L) Not reached (L + R)	HR = 0.59; 95% CI = 0.41 to 0.85; *p* = 0.002
MONALEESA 3	Fulvestrant (F)	Ribociclib (R)	12.8 months (F) 20.5 months (F + R)	HR = 0.593; 95% CI = 0.48–0.73; *p* = 4.10 × 10^–7^
MONALEESA 7	Nonsteroidal AIor Tamoxifen + OFS (ET + OFS)	Ribociclib (R)	13 months (ET) 23.8 months (ET + R)	HR = 0.553 (95% CI = 0.441–0.694; *p* = 9.83 × 10^–8^
MONARCH 3	Nonsteroidal AI (AI)	Abemaciclib (A)	14.7 months (ET) Not reached (ET + A)	HR = 0.54; 95% CI = 0.41 to 0.72; *p* = 0.00021

Abbreviations: PFS (progression free survival); HR (hazard ratio).

**Table 2:** CDK4/6 inhibitors in the second-line setting.

Clinical Trial	Endocrine Agent	CDK4/6 Inhibitor	PFS	HR
PALOMA 3	Fulvestrant	Palbociclib (P)	3.8 months (F) 9.2 months (F + P)	HR = 0.42; 95% CI = 0.32 to 0.56; *p* < 0.001
MONALEESA 3	Fulvestrant (F)	Ribociclib (R)	12.8 months (F) 20.5 months (F + R)	HR = 0.593; 95% CI = 0.48–0.73; *p* = 4.10 × 10^–7^
MONARCH 2	Fulvestrant	Abemaciclib (A)	9.3 months (F) 16.4 months (F + A)	HR = 0.553; 95% CI = 0.449 to 0.681 *p* < 0.001

Abbreviations: PFS (progression free survival); HR (hazard ratio).

**Table 3:** Activity of palbociclib and abemaciclib according to patients’ clinical characteristics.

Substitute for Efficacy Endpoints	PALOMA 2		MONARCH 3	
	Letrozol + Placebo	Letrozol + Palbociclib	AI + Placebo	AI + Abemaciclib
Overall mPFS	14.5 months	24.8 months	14.7 months	Not reached
Liver Metastasis mPFS	8.4 months	13.7 months	7.2 months	15 months
Overall RR	44.4%	55.3%	43.8%	59.2%
Liver Metastasis RR	37%	41.3%	20.7%	54.2%

Abbreviations: mPFS (median progression free survival); RR (response rate); AI (aromatase inhibitor).
